# Genomic Dissection of an Icelandic Epidemic of Respiratory Disease in Horses and Associated Zoonotic Cases

**DOI:** 10.1128/mBio.00826-17

**Published:** 2017-08-01

**Authors:** Sigríður Björnsdóttir, Simon R. Harris, Vilhjálmur Svansson, Eggert Gunnarsson, Ólöf G. Sigurðardóttir, Kristina Gammeljord, Karen F. Steward, J. Richard Newton, Carl Robinson, Amelia R. L. Charbonneau, Julian Parkhill, Matthew T. G. Holden, Andrew S. Waller

**Affiliations:** a MAST Icelandic Food and Veterinary Authority, Reykjavik, Iceland; b Wellcome Trust Sanger Institute, Cambridgeshire, United Kingdom; c Institute for Experimental Pathology, University of Iceland, Reykjavik, Iceland; d University of Copenhagen, Copenhagen, Denmark; e Animal Health Trust, Suffolk, United Kingdom; f University of St. Andrews, Fife, United Kingdom; University of Queensland; University of Minnesota Medical School

**Keywords:** Icelandic horse, *Streptococcus zooepidemicus*, epidemiology, genome analysis, respiratory pathogens, zoonotic infections

## Abstract

Iceland is free of the major infectious diseases of horses. However, in 2010 an epidemic of respiratory disease of unknown cause spread through the country’s native horse population of 77,000. Microbiological investigations ruled out known viral agents but identified the opportunistic pathogen *Streptococcus equi* subsp. *zooepidemicus* (*S. zooepidemicus*) in diseased animals. We sequenced the genomes of 257 isolates of *S. zooepidemicus* to differentiate epidemic from endemic strains. We found that although multiple endemic clones of *S. zooepidemicus* were present, one particular clone, sequence type 209 (ST209), was likely to have been responsible for the epidemic. Concurrent with the epidemic, ST209 was also recovered from a human case of septicemia, highlighting the pathogenic potential of this strain. Epidemiological investigation revealed that the incursion of this strain into one training yard during February 2010 provided a nidus for the infection of multiple horses that then transmitted the strain to farms throughout Iceland. This study represents the first time that whole-genome sequencing has been used to investigate an epidemic on a national scale to identify the likely causative agent and the link to an associated zoonotic infection. Our data highlight the importance of national biosecurity to protect vulnerable populations of animals and also demonstrate the potential impact of *S. zooepidemicus* transmission to other animals, including humans.

## IMPORTANCE

An epidemic of respiratory disease affected almost the entire native Icelandic horse population of 77,000 animals in 2010, resulting in a self-imposed ban on the export of horses and significant economic costs to associated industries. Although the speed of transmission suggested that a viral pathogen was responsible, only the presence of the opportunistic pathogen *Streptococcus zooepidemicus* was consistent with the observed clinical signs. We applied genomic sequencing to differentiate epidemic from endemic strains and to shed light on the rapid transmission of the epidemic strain throughout Iceland. We further highlight the ability of epidemic and endemic strains of *S. zooepidemicus* to infect other animals, including humans. This study represents the first time that whole-genome sequencing has been used to elucidate an outbreak on a national scale and identify the likely causative agent.

## INTRODUCTION

The resident horse population of Iceland is geographically isolated; it arose from animals introduced by settlers in the 9th and 10th centuries, with virtually no import of horses for the last 1,000 years. This isolation of the population has meant that Icelandic horses have remained free from most common contagious diseases of *Equidae*, including equine influenza, equine rhinopneumonitis, equine viral arteritis, *Rhodococcus equi* infection, and strangles (*Streptococcus equi* subsp. *equi* infection) (1–3). As such, the population is extremely vulnerable to these and other infectious agents, and strict biosecurity measures are in place to maintain a high health status.

Between April and November of 2010, an epidemic of respiratory disease in horses that was typified by a persistent dry cough and mucopurulent nasal discharge spread across Iceland, affecting almost the entire population of an estimated 77,000 horses. In addition to the stoppage of all equine activities, it resulted in a self-imposed ban on the export of horses, with important economic consequences for the Icelandic horse industry.

Here, we describe how the opportunistic bacterial pathogen *Streptococcus equi* subsp. *zooepidemicus* (*S. zooepidemicus*) was identified as the causative agent of the respiratory disease and how, by analyzing the genome sequences of *S. zooepidemicus* isolates, a distinct rapidly expanding clone was identified. Furthermore, we demonstrate that this clone was responsible for zoonotic infections during the course of the epidemic and that this strain has become endemic within the Icelandic horse population.

## RESULTS

### Epidemiological network analysis identifies an infection source.

The Veterinary Authority of Iceland received notifications from horse owners and practicing veterinarians indicating cases of the disease all over the country during the spring and summer of 2010. Questionnaires were sent electronically to 200 equine premises throughout the country. Data from 180 (90%) of these premises that responded confirmed that the disease was already widespread throughout Iceland by the first week of April 2010, with affected premises in the south, west, and north of Iceland. These data provided evidence that all horses residing at these premises appeared to be susceptible to the infectious agent, with a morbidity of 100% but low mortality. Dry cough was usually the first clinical sign noted, most often coexisting with symptoms of mucopurulent nasal discharge and mild conjunctivitis, although rectal temperature remained normal in most horses. Laryngoscopy revealed laryngitis and inflammation of the upper trachea. The incubation period was between 2 and 4 weeks and the duration of clinical signs varied from 2 to 10 weeks.

**FIG 1:**
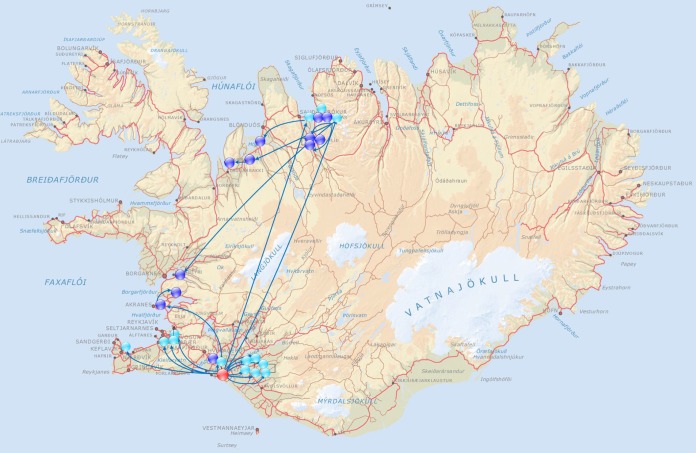
Depiction of the movement of infected horses from the primary center of transmission (red) and the secondary centers of transmission (turkey) to affected farms (blue) during February and March 2010.

The first cases of the epidemic were reported to the Veterinary Authority of Iceland on 7 April by an equine center in the north of Iceland (farm 16). At that time, 10 out of the 42 resident horses showed clinical signs of respiratory tract infection, one of which had been coughing for 16 days. Three other horses at farm 16 had a history of similar clinical signs 2 to 6 weeks earlier. Network analysis of affected farms identified a single common training yard (yard A) as a primary center of transmission (Fig. 1). Two horses leaving yard A on 19 February transmitted the disease both to farm 16 and to a stable in the Reykjavik area. All horses (*n* = 20) that left yard A in March transmitted the disease to new premises (*n* = 18). These new premises each had up to 50 horses stabled and became secondary centers of transmission. However, two horses from yard A which had returned to farm 16 on 4 February were not incubating the disease. Therefore, it is likely that the transmission of the infectious agent to horses at yard A began between 5 and 19 February 2010.

### Identification of *S. zooepidemicus* as a potential causative agent.

The initial assumption due to the rate of spread was that a virus was responsible. Nasal swabs were tested by PCR for viruses known to cause respiratory disease in horses and some common respiratory viruses of humans and animals. Paired blood samples were used to determine if horses developed an antibody response to equine respiratory viruses during the course of their disease. Established and primary cell lines were also used toward the isolation of a potential causal virus. All of these tests proved negative, with the exception of equine gammaherpesviruses, which were isolated from small numbers of both healthy and clinically affected horses (see Table S1 in the supplemental material). Therefore, these viruses were not regarded as being related to the epidemic.

10.1128/mBio.00826-17.2 TABLE S1  Summary of the virological tests employed and results obtained. Download TABLE S1, XLSX file, 0.02 MB. Copyright © 2017 Björnsdóttir et al. 2017 Björnsdóttir et al. This content is distributed under the terms of the Creative Commons Attribution 4.0 International license.

In the absence of a viral pathogen, it was noted that the Gram-positive bacterium *S. zooepidemicus* was isolated from almost all of the nasal swabs taken from coughing horses and from the diseased tissues of occasional fatal cases (Table S2). Initially this was not considered of relevance, because although *S. zooepidemicus* is an opportunistic pathogen that is associated with a range of equine infections (4, 5) and infections of other animals, including humans (6, 7), it is routinely isolated from healthy horses and is widely considered a commensal.

10.1128/mBio.00826-17.3 TABLE S2  Metadata for the *S. zooepidemicus* isolates included in the study and accession numbers for the sequencing data. Download TABLE S2, XLSX file, 0.1 MB. Copyright © 2017 Björnsdóttir et al. 2017 Björnsdóttir et al. This content is distributed under the terms of the Creative Commons Attribution 4.0 International license.

### The population structure of Icelandic *S. zooepidemicus* isolates is dominated by a few dominant clones.

To determine if the epidemic was associated with the introduction and spread of a specific *S. zooepidemicus* strain, we employed whole-genome sequencing (WGS) to interrogate the relationships of 305 *S. zooepidemicus* isolates. A total of 257 isolates were recovered during 2010 from 100 horses residing on 31 different premises across Iceland, and also from two cats, one dog, and three people. Ten archived Icelandic isolates of *S. zooepidemicus* from seven horses, two sheep, and a dog were included to provide an insight into the identity of historical isolates of *S. zooepidemicus* from Iceland. Thirty-eight isolates that were representative of the wider population diversity of *S. zooepidemicus* beyond Iceland were also included in our analysis (Table S2).

**FIG 2:**
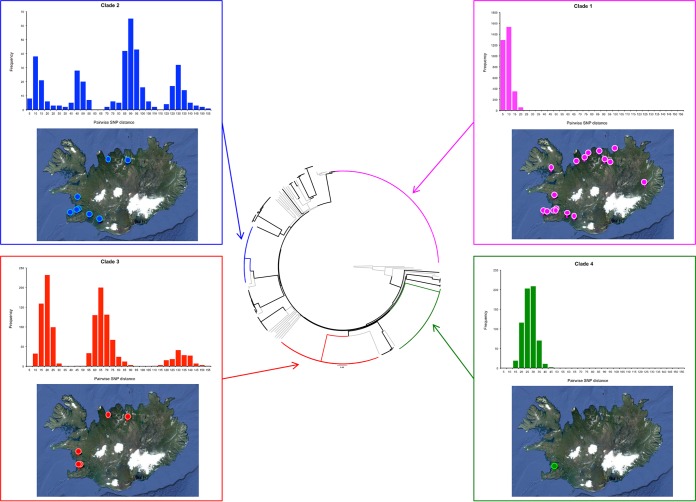
Diversity of the Icelandic *S. zooepidemicus* population. (Center) Phylogenetic reconstruction of the Icelandic *S. zooepidemicus* population structure. The neighbor joining phylogenetic tree was built using core SNPs with SNPs in regions of recombination removed. Included in the phylogeny were *S. zooepidemicus* isolates from outside Iceland that were representative of the genetic diversity of the species. The branches of the tree containing these isolates are shown in gray. The four main clades in the Iceland population, clades 1 (ST209), 2 (ST246), 3 (ST248), and 4 (ST306), are shown in magenta, blue, red, and green, respectively. For each of the clades, the distribution of the pairwise SNP distances calculated for the core genome are displayed (top graph), as is the geographic distribution of the isolates’ origins within Iceland (bottom image). Maps were created using www.spatialepidemiology.net.

Phylogenetic analysis of the WGS data provided a high-resolution view of the Icelandic *S. zooepidemicus* population structure. The majority of Icelandic *S. zooepidemicus* isolates recovered during the epidemic (199 of 257 isolates [77%]) fell into four distinct clades (Fig. 2) that corresponded to four separate multilocus sequence typing (MLST) sequence types (STs). Clade 4 (ST306) contained 37 isolates obtained from eight horses from the Institute for Experimental Pathology, Icelandic Veterinary Institute at Keldur, Reykjavik, that were sampled over a 50-day period from 13 September to 1 November 2010. Six of these horses had signs of respiratory disease over the sampling period. Clade 3 (ST248) contained 52 isolates obtained from 14 horses and a dog. The horses were resident at 8 different farms, and the isolates were recovered over a 160-day period between 19 May and 25 October 2010. Twelve of the horses had signs of disease, including two with pyrexia. The remaining two horses were both from farm 17, had died with signs of interstitial pneumonia identified postmortem, and were infected with ST248 or both ST248 and ST209 (Table S2). Clade 2 (ST246) contained 28 isolates obtained from 14 horses residing at 10 farms over a 162-day period between 6 May and 14 October 2010 and also one human isolate. Two of the horses infected with ST246 had died with evidence of pneumonia or interstitial pneumonia at postmortem examination. All 12 of the remaining horses had clinical signs of disease, including 2 with pyrexia at the time of sampling. Finally, clade 1 (ST209) contained 81 isolates from 45 horses residing at 21 premises across Iceland over a 152-day period between 10 May and 8 October 2010. ST209 was also recovered from a human and a cat. Forty-three of the 45 horses from which ST209 isolates were recovered had clinical signs of respiratory disease at the time of sampling (*n* = 35) or had died (*n* = 8). *S. zooepidemicus* was thought to have contributed to the death of six of these horses, with signs of sepsis, bronchopneumonia, pleuropneumonia, or interstitial pneumonia apparent at postmortem examination. The remaining 58 isolates of *S. zooepidemicus* were recovered from 36 horses, a human, and a cat. Mixed populations of two different isolates of *S. zooepidemicus* were recovered from 13 horses, and three different isolates were recovered from 2 horses.

**FIG 3:**
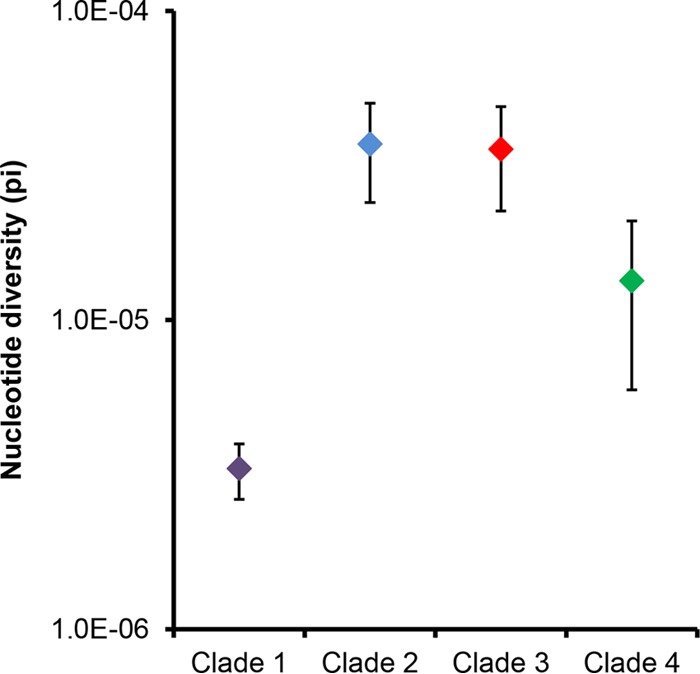
Nucleotide diversity (π) of the four clades of *S. zooepidemicus* recovered from Icelandic horses. Error bars indicate 99% confidence intervals.

The genetic variation within each of the four clades, with duplicate isolates from the same horse removed (Table S2), was summarized using their nucleotide diversity (8), π, calculated using the DendroPy Python library (9). ST209 isolates from 45 Icelandic horses on 21 farms differed by a maximum of 25 single nucleotide polymorphisms (SNPs) (Fig. 2), with a π value of 3.31e−6 (99% confidence interval, 2.63e−6 to 3.98e−6) (Fig. 3). Clade 2 (ST246) isolates from 14 horses at 10 farms differed by a maximum of 153 SNPs, with a π of 3.71e−5 (99% confidence interval, 2.40e−5 to 5.03e−5). Clade 3 (ST248) isolates from 14 horses at 8 farms differed by a maximum of 151 SNPs with a π of 3.57e−6 (99% confidence interval, 2.25e−5 to 4.90e−5). Finally, clade 4 (ST306) isolates from 8 horses at the Icelandic Veterinary Institute differed by a maximum of 44 SNPs, with a π of 1.34e−5 (99% confidence interval, 5.95e−6 to 2.09e−5).

The ST209 isolates of clade 1 had significantly less nucleotide diversity than was identified in the other clades (Fig. 3), but they were recovered from 45 horses at 21 farms which were located over a wide geographic area (Fig. 2). Our data indicate that this strain had been transmitted throughout Iceland over a short period of time, providing evidence that this strain was the most likely cause of the epidemic of respiratory disease.

### ST209 was transmitted from diseased animals and led to disease in previously healthy horses.

Three healthy horses (horses 16, 17, and 18) from the University of Iceland were transferred into a barn (farm 5) on day 0. The 17 resident horses at farm 5 had been diagnosed with respiratory disease on day −14, and *S. zooepidemicus* was recovered from the nasal swabs of 10 horses, which were sampled on day −12. Eight of these 10 isolates were subsequently identified as ST209 by genome sequencing. Following their introduction, the three healthy horses were monitored for clinical signs of disease. Nasal discharge was apparent from day 10, and the discharge became mucopurulent on day 19 and was accompanied by the first observation of coughing. Horse 17 was euthanized on day 23, while horses 16 and 18 were euthanized on day 30 postintroduction. Postmortem examination of these three horses revealed signs of respiratory tract infection which included the presence of mucopurulent material in the nasal cavity, larynx, and trachea and enlarged cervical and mandibular lymph nodes. Histopathological analysis identified subacute rhinitis, laryngitis, and tracheitis with transmigration of neutrophils through the mucosal epithelium. Seven of eight isolates of *S. zooepidemicus* that were recovered on day 28 or during postmortem examination of horses 16 and 18 on day 30 were ST209 (Table S2). However, none of the seven isolates recovered from horses 16, 17, or 18 prior to day 28 were ST209, and the ST209 strain was not recovered from the postmortem examination of horse 17 on day 23 postintroduction. The failure to recover ST209 from horse 17 may have been due to the onset of clinical signs in this animal and its being euthanized on day 23, before shedding of ST209 had accumulated to detectable levels in the presence of other strains of *S. zooepidemicus*. The sampling regimen was, at the time, designed to detect a viral agent and not *S. zooepidemicus*. A greater depth of bacterial isolates recovered from clinical samples may have enabled the subsequent detection of ST209 in a mixed population of *S. zooepidemicus*.

**FIG 4:**
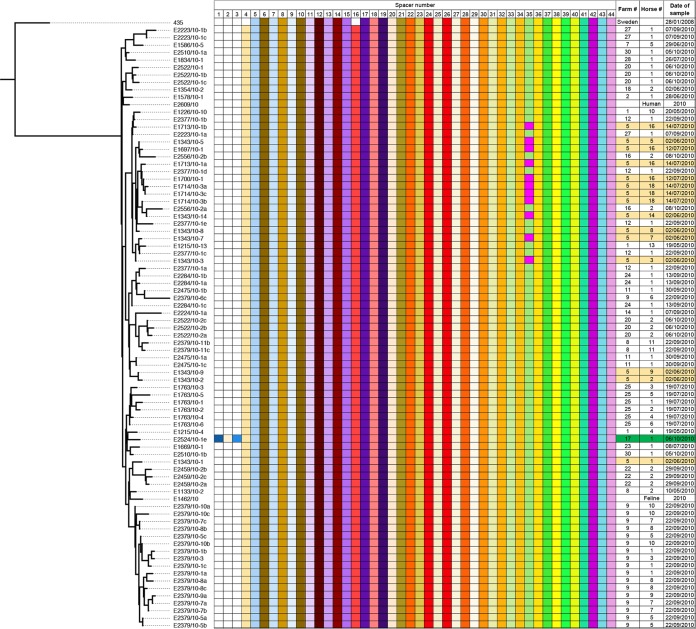
Representation of shared spacer sequences within the CRISPR region of epidemic ST209 isolates relative to the 435 strain that was recovered from Sweden in 2008. (Left) The ML phylogeny of *S. zooepidemicus*. (Right) The presence of shared spacer sequences, from 1 to 44, is indicated by colored boxes, with the date of isolation, farm, and horse number shown in the columns on the right side of the chart. The location of isolates recovered from farm 5 is highlighted in the right panel. An isolate from farm 17 that contained an additional three spacers in its genome sequence is highlighted in green in the right panel.

The very low levels of diversity between ST209 isolates presented a challenge to confirming that the ST209 strains recovered from horses 16 and 18 had been acquired through transmission from the horses at farm 5. However, ST209 isolates, in common with most other strains of *S. zooepidemicus* (10–12), possess a clustered regularly interspaced short palindromic repeat (CRISPR), which provides a snapshot of a strain’s exposure to mobile genetic elements (11, 12). In keeping with rapid transmission across the population, the Icelandic isolates of ST209 predominantly shared the same complement of 41 spacer sequences (Fig. 4). However, ST209 isolates from resident horses 3, 5, 7, and 14 on farm 5 were found to contain a novel spacer 35 sequence. This unique spacer was not present in those isolates recovered from four other resident horses 1, 2, 8, and 9 at farm 5 or from any other affected horses from the other farms throughout Iceland. The ST209 isolates that were recovered from horses 16 and 18 from day 28 contained the unique spacer 35 sequence, directly linking their infection with this strain to their arrival and exposure to the resident horses at farm 5. Our data show that ST209 was transmitted from horses with signs typical of the respiratory disease epidemic to healthy horses and provide further evidence that ST209 was the most likely cause of the epidemic of respiratory disease in Iceland.

### Estimation of the date of common ancestry for the Icelandic ST209 isolates.

**FIG 5:**
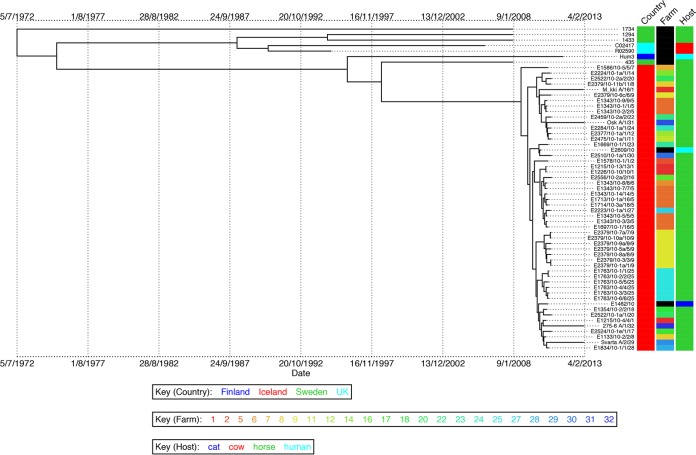
Bayesian phylogenetic visualization of isolate metadata produced using BEAST with replicate samples removed. Branch lengths represent time, with dates shown beneath the tree. Country, farm, and host of origin are indicated in the columns adjacent to the tree.

Following the resolution of the epidemic, we hypothesized that the ST209 strain had become endemic within the Icelandic horse population and that the genetic variation that had accrued since the epidemic could be exploited to estimate the time of most recent common ancestor (TMRCA) shared by the Icelandic ST209 population. To determine if the ST209 strain persisted within the Icelandic horse population, we sampled an additional 36 healthy horses 3 years after the epidemic. A total of 175 *S. zooepidemicus* isolates were recovered. Of these, 20 isolates were identified by quantitative PC (qPCR) as ST209 and had been obtained from four healthy horses stabled at different farms throughout Iceland (Table S2). WGS of the isolates from each of the individual horses indicated that they were very closely related. However, isolates from the different horses clustered separately, with longer branch lengths relative to the isolates recovered from the epidemic in 2010. We analyzed a subset of our data set, for which the date of isolation was known, with the Bayesian phylogenetics software BEAST (13). Removal of strains with identical sequences that were recovered from the same animal on the same date produced a data set of 59 ST209 isolates with 434 polymorphic core genome positions (Table S2). This data set comprised 4 isolates from 2013, 48 isolates that were recovered during the epidemic in 2010, and 7 non-Icelandic isolates which shared an identical or closely related ST to ST209. BEAST includes a number of relaxed molecular clock models that permit modeling of variations in substitution rates on different branches of the tree, allowing correction for the observed rate variation in our data. Utilizing these models, we found that a strict clock with a skyline population model was the best fit of our data, and the mean substitution rate per core genome site per year was calculated as 2.0 × 10^−6^. This substitution rate is similar to core genome rates reported for other streptococci, including *Streptococcus pyogenes* (1.1 × 10^−6^) (14) and *Streptococcus pneumoniae* (1.6 × 10^−6^) (15), and many other Gram-positive bacteria, including *Staphylococcus aureus* (3.3 × 10^−6^) (16). Interestingly, this rate is faster than that for the closely related host-restricted pathogen *Streptococcus equi* (5.2 × 10^−7^) (17), with which *S. zooepidemicus* shares >97% DNA identity (11). This difference most likely reflects the unusual lifestyle of *S. equi*, which can persist within the guttural pouches of recovered horses (17). The analysis provided a median estimate for the TMRCA of the Icelandic ST209 strains of July 2008 (95% highest posterior density, August 2007 to May 2009) (Fig. 5).

## DISCUSSION

This study represents the first time that WGS has been used to investigate an epidemic on a national scale to identify the causative agent. The analysis of WGS data of *S. zooepidemicus* isolates collected during the 2010 epidemic of equine respiratory disease enabled the resolution of recent events from distant transmission events and the identification of the ST209 strain, which was the most likely cause of the epidemic. Our data fully support the epidemiological analysis of this epidemic and point to the incursion of a novel strain of *S. zooepidemicus*, ST209, into the Icelandic horse population. We revealed the diverse pathogenomic properties of *S. zooepidemicus* and how a novel strain can spread rapidly through a susceptible population devoid of sufficient cross-protective immunity despite a background of concomitant colonization with endemic strains.

The endemic strains of *S. zooepidemicus* recovered from Icelandic horses in this study are also likely to have caused clinical signs of disease. ST246 strains were recovered from two fatal cases of pneumonia at farms 21 and 29 in different areas of Iceland, and ST248 strains were recovered from two horses that died from pneumonia at farm 17 (one of which was coinfected with ST209). However, ST246 or ST248 isolates were each recovered from only 14 horses on 8 and 10 farms, respectively. In comparison, ST209 isolates were recovered from 45 horses that were sampled on 21 farms across Iceland. The genetic diversity of ST246 and ST248 strains was significantly higher than that of ST209, suggesting that these other strains were endemic within the Icelandic horse population and were not likely to be responsible for the widespread occurrence of respiratory signs associated with the epidemic in 2010. ST306 strains were only recovered from 8 horses at the Icelandic Veterinary Institute. Even then, the nucleotide diversity of these isolates was significantly higher than that of ST209 isolates. Our data suggest that ST306 had persisted within this closed population of horses for several years and that it was highly unlikely to have caused the epidemic of respiratory disease in 2010.

*S. zooepidemicus* can cause severe disease in humans (6, 7, 18–20) and other animals (21–23), and transmission from animals to humans has been demonstrated (6, 19). The epidemic ST209 strain was recovered from a cat and from a blood sample from an Icelandic woman who had suffered a miscarriage (E1462/10 and E2609/10, respectively) (Fig. 5). A closely related strain of *S. zooepidemicus* ST209, Hum3, was recovered from a psoas abscess in a Finnish man in 2011 (24), suggesting that ST209 strains of *S. zooepidemicus* may have increased potential to cross host boundaries to cause zoonotic disease. Several other cases of human infections in Iceland during 2010 may have been caused by the ST209 strain, but unfortunately these isolates were not available for sequencing. The other two isolates of *S. zooepidemicus*, E2828/10 and E2827/10, that were recovered from humans during the epidemic period were ST246 and ST2, respectively. Both of these STs are representatives of endemic Icelandic strains, highlighting that the prevalence of zoonotic transmission of *S. zooepidemicus* may be underreported.

Yard A opened in January 2010 and offers rehabilitation for up to 35 horses recovering from injury or poor condition as well as equipment training for competition horses. Although the epidemic agent was generally transmitted through the coughing of infected horses, coughing was not observed at yard A before April 2010, most probably due to a relatively long incubation period permitting apparently healthy horses to return to their original premises posttraining. Instead, the most likely route of transmission of the epidemic strain at yard A was a water treadmill, which horses used on a daily basis. The water used in the treadmill contained no disinfectant and was changed on a once- or twice-weekly basis, providing ideal conditions for the transmission of *S. zooepidemicus* between the visiting horses. The addition of chlorine coupled with regular cleaning and disinfection of water treadmills may minimize or eliminate the transmission of *S. zooepidemicus* or other infectious agents via this route.

The TMRCA for the Icelandic isolates was estimated to be July 2008. However, we note that ST306 isolates recovered from eight horses stabled at the Icelandic Veterinary Institute over a period of 50 days had significantly higher nucleotide diversity than the ST209 isolates, which were recovered from 45 horses throughout Iceland over a much longer period of time. Our data suggest that the ST306 isolates of *S. zooepidemicus* likely persisted in the horses at the Icelandic Veterinary Institute, accruing nucleotide diversity over time within this isolated population. Analysis of *S. equi* isolates recovered from persistently infected animals identified that they had an increased rate of substitution and that isolates from the same animal could accrue substitutions, amplifications, and deletions (17). Therefore, it is possible that a mixed population of ST209 variants, which could have originated from one or more horses, may have been introduced to Iceland at a date much closer to the start of the 2010 epidemic. However, it is also possible that the proposed transmission of ST209 strains, for example, via the submerged treadmill at yard A, provided conditions that led to a selective sweep of a more diverse population of ST209, with the successful variants establishing the epidemic. In this manner, the introduction of ST209 into Iceland may have predated the calculated TMRCA.

The Icelandic epidemic ST209 strains share greatest genetic similarity to strain 435, which was isolated from a coughing horse in Sweden during 2008. To date, with the exception of Iceland, ST209 strains have only been isolated from Scandinavia, and it is interesting the close relationships between many training yards in Iceland and this region of Europe. The import of horses to Iceland has been prohibited since 1882 for biosecurity reasons. Although the import of used riding equipment is also prohibited, it is difficult to control. Therefore, contaminated tack represents a possible industry-related route of introduction of the ST209 strain into Iceland. The ability of ST209 strains to cross host boundaries provides an alternative import mechanism whereby the infection of a human may have facilitated onward anthroponotic transmission to horses resident in Iceland. While it is not possible to identify the precise route by which the ST209 strain entered Iceland, a heightened awareness of the routes of transmission will greatly reduce the risk of future epidemics of disease in this important horse population.

## MATERIALS AND METHODS

### Epidemiological investigation.

A questionnaire was sent to 200 premises, including all professional training yards and breeding farms in the country, in June 2010 and September 2010 to determine whether and when horses had shown signs of respiratory disease. Further information pertaining to the movement of horses into affected premises was collected by interviews, enabling the network of connected farms and training centers to be investigated.

### Contact study.

Three clinically healthy Icelandic horses (horses 16, 17, and 18), aged 18 to 21 years, from the Institute for Experimental Pathology at Keldur were transferred to farm 5 2 weeks after the first signs of respiratory disease were identified in the resident horses. The experiment was conducted in accordance with the Icelandic animal care guidelines for experimental animals (the Icelandic Animal Welfare Act 15/1994), the Icelandic Regulations on Animal Experimentation 279/2002, European Treaty series 123, 18.III.1986, and after formal approval from the Icelandic Ethical Committee on Animal Research, license 0710-0401. The horses were examined daily for the onset of clinical signs. Nasal swabs (Coban) for bacteriological and virological examination and blood samples, serum and with EDTA (Vacuette), were collected every 2 days.

### Postmortem examinations.

For postmortem examinations, samples were taken from all major organs in addition to samples from the nasal mucosa, larynx, and trachea. Tissues were fixed in 10% neutral buffered formalin. The formalin-fixed material was embedded in paraffin, and 4-µm-thick sections were cut, mounted, and stained with hematoxylin and eosin.

### Virus isolation and diagnostics.

Nasal swabs and enriched peripheral blood leukocyte cells (PBLC) in plasma from EDTA-stabilized blood samples from diseased and healthy animals were used to inoculate retroviral vector LXSN116E6E7-transfected fetal lung and kidney cell lines (L. Thorsteinsdóttir et al*.*, unpublished data), primary equine fetal lung and kidney cells (3), RK-13 cells (ATCC), MA-104 cells (Microbiological Associates), and Vero cells (ATCC) toward the isolation of viral particles.

Serological tests and PCR assays for the detection of equine herpesvirus types 1, 2, 4, and 5 equine infectious anemia virus, equine arteritis virus, equine rhinitis virus A and B, equine influenza virus A type 1 (H7N7) and type 2 (H3N8), equine salemvirus, human and equine adenovirus, mammalian reovirus serotypes 1, 2, and 3, carnivore parvovirus, human rhinovirus, human enterovirus, canine pneumovirus, influenza virus A and B, parainfluenza virus 3, and mammalian coronaviruses were used according to the methods in the *Manual of Diagnostic Tests and Vaccines for Terrestrial Animals* produced by the OIE and other published methods (3, 25–40).

### Study isolate collection.

The origins and details of the 305 isolates of *S. zooepidemicus* that were included in this study are listed in Table S2. A total of 251 isolates were recovered from the nasal swabs of 100 horses that were actively showing signs of respiratory disease or were stabled on 31 affected premises during the epidemic. Multiple isolates recovered from the same clinical sample or the same horse sampled over time optimized the isolation of the epidemic strain in the face of concomitant colonization. Six Icelandic isolates recovered from cases of *S. zooepidemicus* disease in companion animals (two cats and one dog) and in three people that occurred during the epidemic, and Icelandic isolates from seven horses, one dog, and two sheep that predated the 2010 epidemic were included in the analysis. Ten archived Icelandic isolates of *S. zooepidemicus* from seven horses, two sheep, and a dog were included to provide an insight into the identity of historical isolates of *S. zooepidemicus* from Iceland. We also included the sequenced genomes of 38 strains of *S. zooepidemicus* from outside Iceland that were representative of the known species diversity as a whole, as defined by MLST (41), including the published genome sequences for *S. zooepidemicus* strains MGCS10565 (10), H70 (11), BHS5 (42), and ATCC 35246 (43).

### DNA sequencing.

DNA was purified from *S. zooepidemicus* isolates by using a GenElute column kit as described previously (41). DNA libraries for isolates recovered pre-2011 were created using a method adapted from the Illumina indexing standard protocol. Genomic DNA was fragmented by acoustic shearing to enrich for 200-bp fragments by using a Covaris E210, end repaired, and A tailed. Adapter ligation was followed by overlap extension PCR using the Illumina 3 primer set to introduce specific tag sequences between the sequencing and flow cell binding sites of the Illumina adapter. DNA was quantified by qPCR and sequenced on the Illumina GAII and HiSeq platforms according to the manufacturer’s protocols, generating index tag end sequences. For isolates recovered post-2011, DNA libraries were prepared using the Illumina Nextera XT DNA library prep kit, according to the standard protocol. The DNA libraries were pooled and quantified using the Kapa library quantification kit for Illumina platforms prior to sequencing on Illumina MiSeq according to the manufacturer’s protocol.

### Variation detection.

Illumina reads were mapped onto the relevant reference sequences by using SMALT (http://www.sanger.ac.uk/resources/software/smalt/), with H70 (GenBank accession number FM204884) for the total population and a *de novo* assembly of clade 1. A minimum 30-fold depth of coverage for more than 92% of the reference genomes was achieved for both reference sequences (Table S2). The default mapping parameters recommended for reads were employed but with the minimum score required for mapping increased to 30 to make the mapping more conservative. Candidate SNPs were identified using SAMtools mpileup (44), with SNPs filtered to remove those at sites with a mapping depth of less than 5 reads and an SNP score below 60. SNPs at sites with heterogeneous mappings were filtered out if the SNP was present in less than 75% of reads at that site (16). Identification of the core genomes was performed as previously described (16, 45). Recombination was detected in the genomes by using Gubbins (http://sanger-pathogens.github.io/gubbins/) (46). Figure S1 summarizes the Gubbins output for clade 1 (ST209) isolates. Phylogenetic trees were constructed separately using RAxML v7.0.4 (47) for all sites in the core genomes containing SNPs, with a general time-reversible (GTR) model with a gamma correction for among-site rate variations (16). The genetic variation within each of the four clades, with isolates from duplicate horses removed (Table S2), was summarized using their nucleotide diversity (8), π, calculated using the DendroPy Python library (9). The variances of the nucleotide diversity estimates were calculated as described in reference 48 and used to compute confidence intervals using a normal distribution approximation. We used the Bayesian software package BEAST (v1.7.4) (49) to investigate the temporal, spatial, and demographic evolution of the ST209 population, with replicate samples removed from the analysis. To estimate the substitution rates and times for divergences of internal nodes on the tree, a GTR model with a gamma correction for among-site rate variation was used. All combinations of strict, relaxed log normal, relaxed exponential, and random clock models and constant, exponential, expansion, logistic, and skyline population models were evaluated. For each, three independent chains were run for 100 million generations, sampling every 10 generations. A maximum clade credibility tree was created from the resulting combined trees created using the treeAnnotator program, also from the BEAST package.

10.1128/mBio.00826-17.1 FIG S1  Representation of predicted homologously recombined regions in clade 1 (ST209 and closely related strains). (Left) The ML phylogeny of the clade 1 strains of *S. zooepidemicus*. (Right) Regions identified as exhibiting a significantly raised SNP density, indicative of import of variation en masse via homologous recombination or regions under high selective pressures. Download FIG S1, PDF file, 0.02 MB. Copyright © 2017 Björnsdóttir et al. 2017 Björnsdóttir et al. This content is distributed under the terms of the Creative Commons Attribution 4.0 International license.

### ST209-specific qPCR.

ST209 isolates recovered in 2013 were initially identified using a specific qPCR for *nrdE* allele 31 prior to genome sequencing. Forward (5′-ACCAAAAAAGAAAATGCT-3′) and reverse (5′-TCAACACTATAAGGACTAAAGAGA-3′) primers were used to amplify *nrdE* allele 31 on a StepOnePlus machine (Applied Biosystems) with Sybr Fast ABI Prism reagents (Kapa Biosystems) and cycling conditions of 95°C for 3 min followed by 40 cycles of 95°C for 30 s and 62°C for 10 s. All qPCR experiments were performed in triplicate.

### Accession number(s).

The Illumina sequences generated and used in this study have been deposited in the European Nucleotide Archive under the accession number ERP000883. The accession numbers for the sequences of each isolate are listed in Table S2.
