# Supplementary figures and images for: Genomic Dissection of an Icelandic Epidemic of Respiratory Disease in Horses and Associated Zoonotic Cases
Source: mBio. 2017 Aug 1;8(4):e00826-17. doi: 10.1128/mBio.00826-17 (PMC5539424; doi:10.1128/mBio.00826-17)

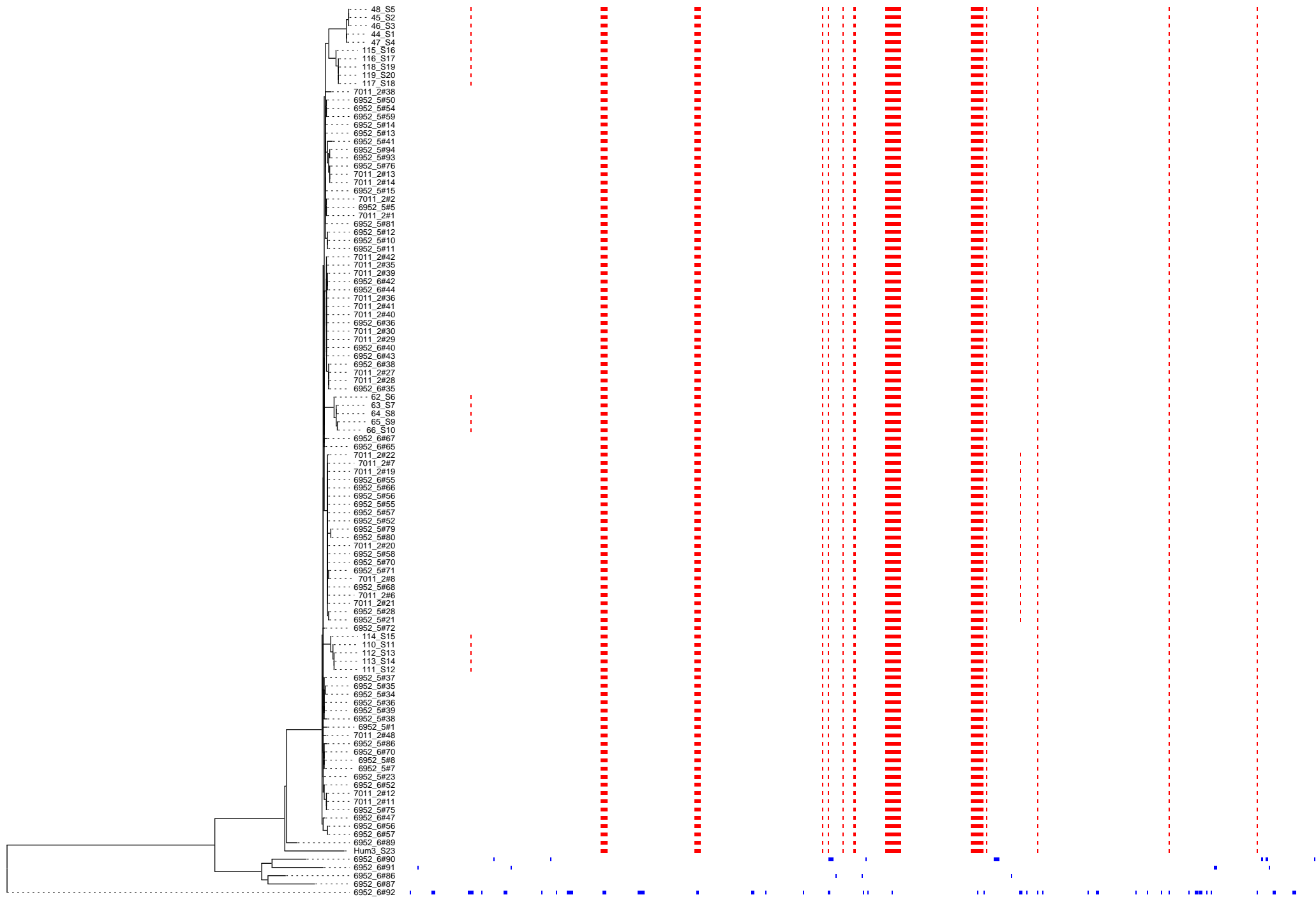

0.059

Supplement: FIG S1 [file mbo004173408sf1.pdf]
